# HIV, Viral Hepatitis, and Schistosomiasis Association with Liver Cancer: A Systematic Review

**DOI:** 10.3390/microorganisms13122753

**Published:** 2025-12-04

**Authors:** Khumbuzile Canham, Pragalathan Naidoo, Sibusiso Senzani, Sayed Shakeel Kader, Zilungile L. Mkhize-Kwitshana

**Affiliations:** 1 Department of Medical Microbiology, School of Laboratory Medicine and Medical Sciences, College of Health Sciences, University of KwaZulu-Natal, Nelson R. Mandela Medical School Campus, Durban 4001, South Africa; naidoop5@ukzn.ac.za (P.N.); senzanis@ukzn.ac.za (S.S.); 2 Division of Research Capacity Development (RCD), South African Medical Research Council (SAMRC), Tygerberg, Cape Town 7505, South Africa; 3 Department of Surgery, University of KwaZulu Natal, Congella, Durban 4001, South Africa; shakeelkader2006@gmail.com; 4 Biomedical Sciences School of Life and Consumer Sciences, College of Agriculture and Environmental Sciences, University of South Africa, Florida Campus, Johannesburg 1710, South Africa; mkhizzl@unisa.ac.za

**Keywords:** hepatocellular carcinoma, hepatitis B, hepatitis C, schistosoma, inflammation

## Abstract

Liver cancer is a notable global health concern, with several infections contributing to its etiology. This systematic review investigates the roles of HIV, viral hepatitis, and schistosomiasis in the development of liver cancer, particularly hepatocellular carcinoma. This systematic review was registered under PROSPERO with the reference: CRD42024566941. A comprehensive literature search was conducted using various databases (PubMed, ScienceDirect, Google Scholar, Scopus, and Web of Science) to identify studies examining the association between HIV, viral hepatitis, and schistosomiasis with hepatocellular carcinoma. The inclusion criteria were studies published in English between the years 2000 and 2025 that primarily explored the association and process through which HIV, viral hepatitis, and schistosomiasis trigger hepatocarcinogenesis. Data retrieval and quality assessment were conducted independently by all co-authors. Overall, 31 studies were deemed relevant to this systematic review. Findings indicate that HIV-associated immunosuppression significantly increases the risk of HCC, with one study reporting a 70% increased incidence among its cohort, and another study noting an 87% increase in HCC-related mortality. Among viral hepatitis cases, one study reported that 86% of HCC infections were attributed to HBV, while HCV genotype 3 was associated with a 68.8% mortality rate in HCC patients. For schistosomiasis, a study showed that 8.1% of schistosomiasis patients with portal vein thrombosis developed HCC. No studies were identified on the association of liver cancer with simultaneous multi-infection by HIV, viral hepatitis, and schistosomiasis. These results underscore the necessity for targeted interventions and integrated strategies to prevent and treat single and concurrent infections that could accelerate infection-associated liver cancer.

## 1. Introduction

Liver cancer remains a major public health concern; globally, it is estimated that over a million individuals will be affected by the year 2025 [1]. Of all the liver cancers that are currently recognized, hepatocellular carcinoma (HCC) is the most prevalent type and accounts for about 90% of all cases [1]. HCC, which makes up about 7% of all cancer cases, constitutes the sixth most frequently detected type of cancer and the third largest cause of cancer-related deaths worldwide [2,3,4]. HCC develops due to the neoplastic conversion of hepatic cells and is one of the most challenging cancers to treat [5]. In 2020, there were 905,677 new cases and 830,180 deaths attributed to this disease globally [2]. HCC is found primarily along the Asia-Pacific coast and Southeast Africa, and also in other regions with low prevalence, including Australia, Europe, and North America [6]. In sub-Saharan Africa, it is a common healthcare hazard that is frequently disregarded, misdiagnosed, or detected late [5]. The aetiology of HCC includes infectious and non-infectious factors. Persistent hepatitis B (HBV) and hepatitis C virus (HCV) infection along with early aflatoxin-B1 (AFB1) exposure are the primary risk factors for HCC in sub-Saharan Africa; however, immoderate alcohol consumption, high levels of iron, and HIV infection may also be contributing factors to the incidence of HCC [7]. Another biologically plausible cause of liver cancer that is mostly under-researched is schistosomiasis. The liver is one of the key sites affected during the life cycle of schistosomiasis, a neglected tropical disease [8]. In the liver, the infection induces chronic inflammation and fibrosis, both of which are recognized as pre-cancerous conditions [8].

### 1.1. Human Immunodeficiency Virus (HIV) and HCC

Liver cancer, especially HCC, is a serious concern among people who are infected with HIV or acquired immunodeficiency syndrome (AIDS). The immunosuppression resulting from HIV infection compromises the body’s ability to identify and eradicate malignant or pre-cancerous cells [9]. HIV mainly targets and destroys CD4+ T cells, which are essential for immune response coordination [10]. As HIV advances, the body loses a remarkable number of these cells, leading to an acute immunological deficiency [10]. Losing these CD4+ T cells impairs the function of other important immune cells, including cytotoxic T lymphocytes and natural killer cells, which depend on signals from CD4+ T cells to become completely stimulated and effective [11]. The cytotoxic T lymphocytes and the natural killer cells detect cells that have undergone mutations or have started acting abnormally [11]. This implies that with the reduced immune monitoring, the pre-cancerous cells can remain undetected for longer periods, allowing them the opportunity to proliferate and become fully developed tumors [12].

Furthermore, HIV viral proteins may interact with hepatocytes, steering the production of pro-inflammatory cytokines or cellular stress responses, potentially leading to liver damage [13]. HIV proteins, including Tat and gp120, have been demonstrated to promote liver fibrosis and dysregulate cell proliferation, fostering a cancer-conducive environment [14]. As liver fibrosis worsens due to chronic HIV protein exposure, the liver’s architecture becomes altered, increasing the likelihood that cells may convert malignantly [15].

Moreover, continuous use of antiretroviral therapy (ART), especially older regimens of therapy, has been associated with hepatotoxicity that may ultimately increase the risk of HCC [16]. HIV, alongside ART, may trigger metabolic syndrome, such as insulin resistance, and non-alcoholic fatty liver disease [17]. The liver fat build-up exacerbated by lipid abnormalities and insulin resistance can advance to a prolonged liver inflammation, leading to HCC development [17]. While liver cancer is more commonly seen in people with HIV who are co-infected with hepatitis B or C, these mechanisms suggest that HIV alone can increase the risk of liver cancer, even in the absence of other viral co-infections.

### 1.2. Viral Hepatitis and HCC

Viral hepatitis is a global health concern, with the widespread types of hepatitis being A, B, C, D, and E [18]. Chronic HBV and HCV infections account for about 91% of hepatitis fatalities [18]. HBV and HCV account for over 80% of HCC occurrences worldwide [19,20]. Sub-Saharan Africa has a high prevalence of HBV infection, with approximately 80 million individuals having a chronic infection [21].

Although chronic infection with either the RNA virus HCV or the DNA virus HBV promotes HCC, the mechanisms by which these viruses cause HCC are different, and the disease presents in clinically diverse patterns in each case [22]. HBV drives the development of HCC through numerous mechanisms, including modifying transcription in cells, controlling replication by the persistent expression of the HBV x protein, and epigenetic changes by the x and core proteins [23]. Once HBV enters the cytoplasm of recipient hepatocytes, it begins to release its partially two-stranded, relaxed-circular DNA (rcDNA), which is transported to the nucleus, forming covalently closed circular DNA (HBV cccDNA), the main blueprint for viral replication [24]. HBV cccDNA acts as a template for creating viral RNA, which is subsequently translated into strong oncogenic proteins, like the HBx protein [25]. The HBx protein partakes in the suppression of tumor suppressors, including protein p53, dysregulation of cell signalling pathways like the STAT3 pathway, DNA damage, genomic disorders, and genetic alterations that fuel oncogenesis [25,26].

On the other hand, HCV replication in hepatocytes causes oxidative stress, leading to the generation of reactive oxygen species (ROS) [27]. These ROS can cause direct DNA damage, interfere with the body’s natural defences, resulting in mutations in tumor suppressor genes and key oncogenes [27,28]. The virus itself disrupts regular mitochondrial activity, further increasing oxidative stress in liver cells [27,28]. Some HCV proteins, like the HCV core protein and non-structural proteins, have been demonstrated to interact with host cell pathways related to immunological evasion, cell proliferation, and apoptosis [29]. These proteins can disrupt normal messenger molecules, encouraging unregulated cell growth and survival, aiding the cancerous transformation of liver cells [29].

### 1.3. Schistosomiasis and HCC

Schistosomiasis is a chronic illness that affects about 240 million people globally [30] and results in over 500,000 fatalities annually [31]. Schistosomiasis, caused by trematodes of the family Schistosoma, is among the most devastating parasitic infections in the world [32]. The infection is common in impoverished tropical and subtropical populations with inadequate sanitation and potable water supplies [30]. After malaria, schistosomiasis is the second most prevalent human parasitic disease in the world [33]. There are five species of Schistosoma, namely *S. japonicum*, *S. hematobium*, *S. mekongi*, *S. mansoni*, and *S. intercalatum* [34]. The majority of infections in humans are caused by *S. japonicum* and *S. haematobium*, which have been closely associated with HCC and carcinoma of the bladder, respectively, and *S. mansoni*, which has been associated with numerous case reports of prostate cancer, liver cancer, giant follicular lymphomas, and colorectal cancer [32,34].

The continuous build-up of parasite eggs in the liver and the immune system response towards them are the primary causes of schistosoma-related liver cancer [35]. Chronic schistosomiasis infection causes a persistent inflammatory response in the liver [36]. The host’s immune system reacts to parasite eggs deposited in the liver’s portal veins, leading to the formation of granulomas and subsequent fibrosis [36,37]. The persistent inflammation and fibrosis lead to the development of HCC [31,38]. While chronic inflammation and fibrosis are key mechanisms linking schistosomiasis to HCC, other factors also play a role. Furthermore, environmental variables such as susceptibility to other carcinogens, dietary practices, and concurrent infections (e.g., viral hepatitis and HIV) can enhance the likelihood of HCC in chronic schistosomiasis patients [39].

It is noted that all three infections, HIV [40], viral hepatitis [41], and schistosomiasis [32], can individually lead to the development of liver cancer since they all directly and indirectly result in the inflammation of the liver. In cases where they all co-exist, it is logical to predict that they can result in elevated chronic liver inflammation, which can influence the onset of liver cancer through genetic mutations, interfering with regular cellular functions, and creating conditions that encourage the growth and metastasis of malignant tumors. However, there are currently no studies that focus on the concurrent infection with all three and their impact on liver cancer development. It is then essential to study the impact of this potential triple infection and its association with liver cancer. To bridge this knowledge gap, this systematic review sought to investigate the association of HIV, viral hepatitis, and schistosomiasis with liver cancer. It is expected that the consolidated data will generate knowledge and emphasis on the combined effects of HIV, viral hepatitis, and schistosomiasis triple infection on liver cancer oncogenesis.

## 2. Materials and Methods

This systematic review collected relevant data from previous literature regarding the association of HIV, viral hepatitis, and schistosomiasis with liver cancer. A narrative approach was followed to review relevant and available data on this topic. Most relevant articles were selected based on the inclusion criteria. This systematic review was registered under PROSPERO (International Prospective Register of Systematic Reviews) with reference: CRD42024566941. PROSPERO is an online database where researchers can register their planned or ongoing systematic reviews to prevent duplication of systematic reviews.

### 2.1. Literature Search Strategy

PubMed, ScienceDirect, Google Scholar, Scopus, and Web of Science databases were utilised to search the following keywords, using the Boolean operators “and” “or”: ‘HIV, “or” Human Immunodeficiency Virus, and Liver Cancer, “or” Hepatocellular Carcinoma’; ‘Viral Hepatitis and Liver Cancer, “or” Hepatocellular Carcinoma’; ‘Schistosoma, “or” Schistosomiasis, “or” Bilharzia, and Liver Cancer, “or” Hepatocellular Carcinoma’. To find appropriate literature, these keywords were incorporated, periodically replacing the word “and” with the term “association with”. The relevant data were screened, analysed, and reported following the PRISMA (Preferred Reporting Items for Systematic Reviews and Meta-Analysis) guidelines. This review included all English-language published literature globally from the year 2000 to the year 2025.

### 2.2. Study Selection and Data Extraction

First, in compliance with the inclusion and exclusion criteria, the primary author (K.C.) and co-author (P.N.) independently selected relevant literature based on titles, abstracts, and full texts, which were read sequentially for screening relevance. An Excel spreadsheet was utilized to independently extract and code the data. The data acquired encompassed basic information of the included studies, including the research title, first author, and year of publication, research type, total number of participants with HIV, viral hepatitis, and schistosomiasis infections.

### 2.3. Inclusion Criteria

▪ Literature reporting on the association of HIV, viral hepatitis, and schistosomiasis with liver cancer, HCC.
▪ Literature published in all countries and in the English language from January 2000 to March 2025.
▪ Literature, including animal and human studies.
▪ Molecular and genetic studies, which include the investigation of the molecular and genetic mechanisms linking HIV, hepatitis, and schistosomiasis to liver cancer development.
▪ Cohort studies—observational studies that follow a group of people over time to see who develops liver cancer after exposure to either HIV, viral hepatitis, or schistosomiasis.
▪ Case-control studies that compare individuals with liver cancer (cases) to those without (controls) to assess past exposure to HIV, hepatitis, or schistosomiasis.
▪ Case series and case reports, retrospective studies.

### 2.4. Exclusion Criteria

Studies were excluded if they:

▪ Were review articles,
▪ Were duplicate studies,
▪ Had incomplete or unclear study information.
▪ Not published in English
▪ Articles published prior to January 2000
▪ Conference abstracts and book chapters

### 2.5. Quality Assessment

The quality of included studies was assessed using the Effective Public Health Practice Project (EPHPP) Quality Assessment Tool for Quantitative Studies. This tool assesses the quality of the studies based on these components: selection bias, study design, confounders, blinding, data collection methods, withdrawals and dropouts, and analysis. Each domain was rated as strong, moderate, or weak, leading to an overall global rating for each study. Assessments were performed independently by the co-authors (K.C., P.N., S.S., S.S.K.), and results were summarized in a tabular form (Supplementary Table S2). Any discrepancies in ratings were discussed and resolved, with final decisions settled by the fifth author (Z.L.M.-K.), who has greater expertise in the study. Studies with more than two weak ratings were generally considered of lower quality and were excluded from this study.

**Figure 1:**
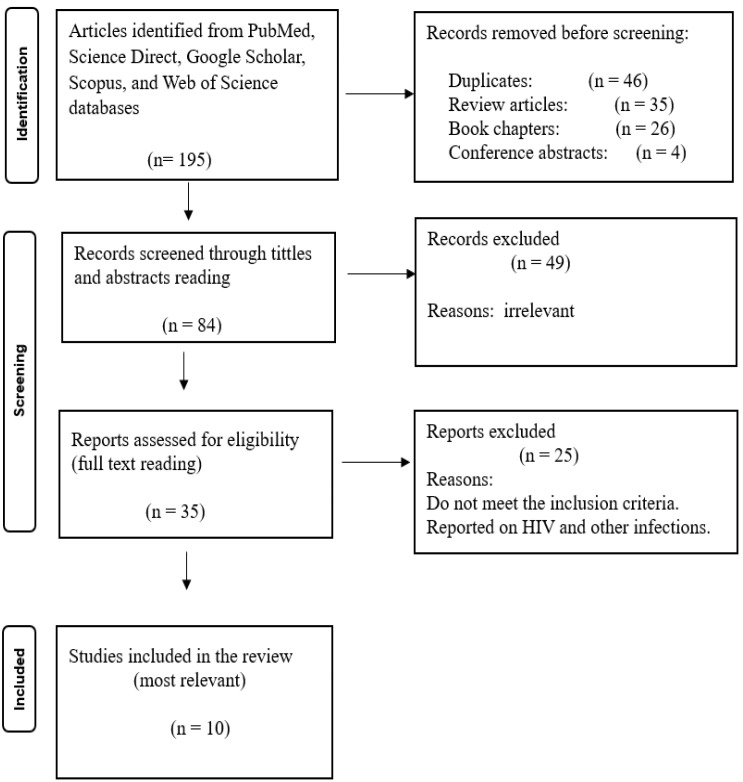
PRISMA flow diagram showing the article selection process used to collect, screen, and identify eligible data of relevant articles based on HIV association with liver cancer (HIV and Liver Cancer).

**Figure 2:**
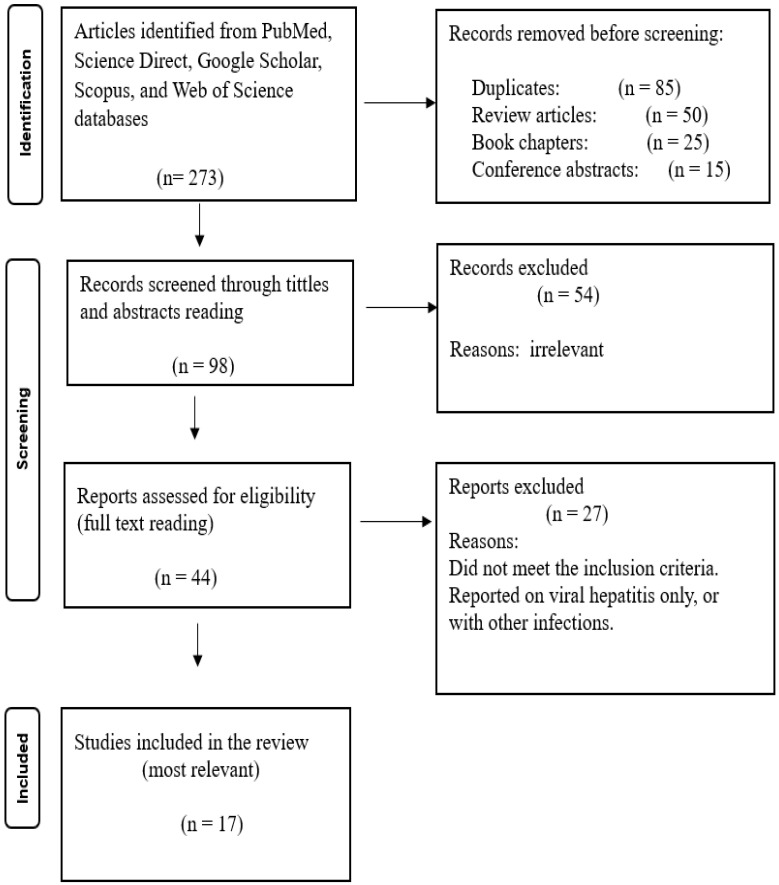
PRISMA flow diagram showing the article selection process used to collect, screen, and identify eligible data of relevant articles based on viral hepatitis association with liver cancer (Viral Hepatitis and Liver Cancer).

**Figure 3:**
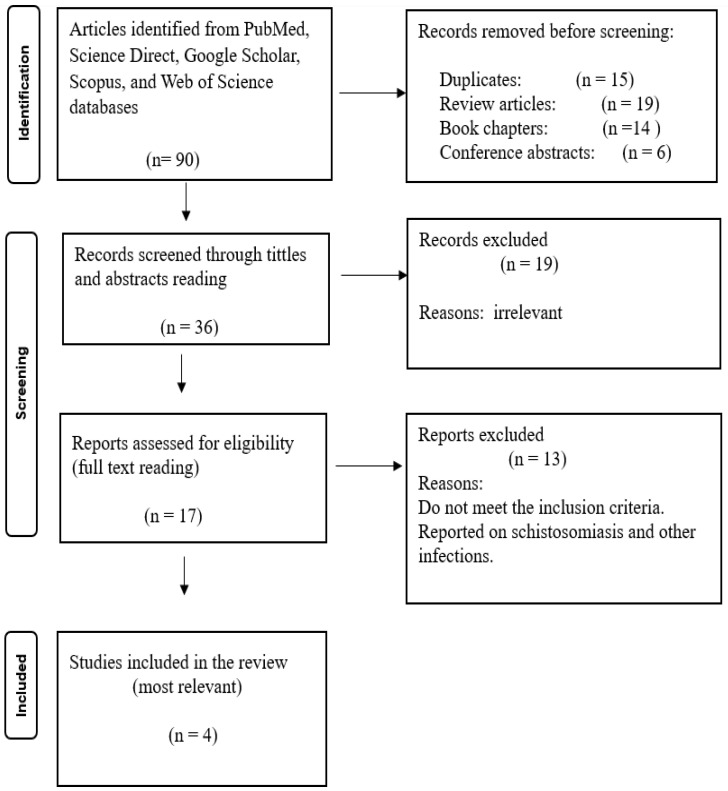
PRISMA flow diagram showing the article selection process used to collect, screen, and identify eligible data of relevant articles based on schistosomiasis association with liver cancer (Schistosomiasis and Liver Cancer).

In this systematic review, a total of 31 articles were included according to the established inclusion and exclusion criteria. The initial search yielded 195 articles on HIV and liver cancer, 273 on viral hepatitis and liver cancer, and 90 on schistosomiasis and liver cancer from the specified search engines. After removing duplicates, review articles, book chapters, and conference abstracts, 84 of the 195 articles on HIV and liver cancer were further evaluated against the eligibility criteria, resulting in 35 reports being assessed for eligibility, with 10 articles deemed most relevant and included in the review. For viral hepatitis and liver cancer, 98 articles remained after deduplication, review articles and book chapters removal, with 44 assessed for eligibility. Following full-text screening, 17 articles were included in the review. For schistosomiasis and liver cancer, 17 articles were screened for eligibility, and 4 were found to be the most relevant and included in the review. Following full-text screening for all articles, most studies were removed because they focused solely on HIV, viral hepatitis, or schistosomiasis, either individually or in combination with other infections unrelated to liver cancer. Since the focus of this review is the association between HIV, viral hepatitis, schistosomiasis, and liver cancer, studies that did not meet this criterion were excluded. No studies were found using the combined keywords “HIV, viral hepatitis, schistosomiasis, and liver cancer,” indicating a lack of published research on the association of liver cancer with the multi-infection of HIV, viral hepatitis, and schistosomiasis. The PRISMA 2020 flow diagrams below (Figure 1, Figure 2 and Figure 3) illustrate the whole selection process.

## 3. Results

**Figure 4:**
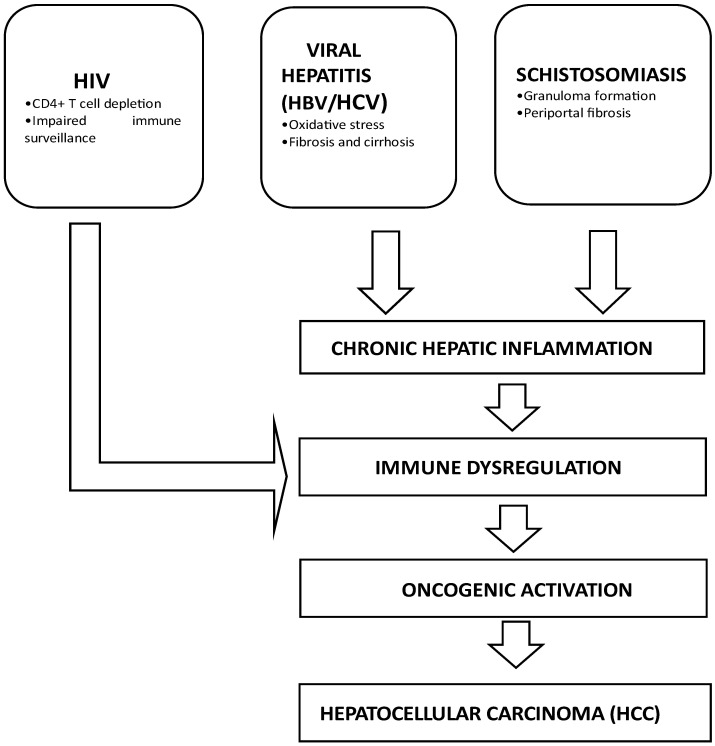
Schematic illustration of the association of three infections -HIV, viral hepatitis, and schistosomiasis with the development of HCC.

The results of the studies included in this systematic review are summarized in three tables. Table 1 presents the studies investigating the association between HIV and liver cancer. Table 2 summarizes the studies exploring the link between viral hepatitis and liver cancer. Lastly, Table 3 outlines the studies investigating the relationship between schistosomiasis and liver cancer. These tables provide a comprehensive overview of the pertinent research findings for each association. Figure 4 shows the schematic illustration of the mechanisms through which HIV, viral hepatitis (HBV/HCV), and schistosomiasis may contribute to the development of HCC, as identified across studies included in this systematic review.

## 4. Discussion

This is the first systematic review that summarised the association of HIV, viral hepatitis, and schistosomiasis with the development of HCC. The summarized studies indicate that HIV is associated with the development of liver cancer, primarily through immune system suppression, while viral hepatitis contributes to liver cancer by inducing chronic inflammation and liver scarring, with an elevated risk when co-infected with HIV. Regarding schistosomiasis, findings suggest that schistosomiasis, through chronic inflammation and fibrosis among others, promotes genetic variation and oxidative stress, which are precursor conditions to liver cancer.

The mechanisms of HIV that lead to the development of HCC are not clear [66]. Nevertheless, research suggests that HIV-induced immunosuppression is linked to an increased likelihood of HCC development [42,45,46]. Studies also show that a decreased CD4+ count is significantly linked to HCC, suggesting a possible direct role for immune suppression in cancer development [67,68,69]. This was made apparent in a recent study by Nsibirwa et al., which examined the effect of HIV infection on the manifestation and prognosis of HCC. They found that patients who had acute HIV-related immunodeficiency (CD4 count < 200 cells per cubic milliliter) had an increased risk of developing HCC [2]. Lu et al. also found that a decreased CD4 count in HIV/HCC co-infection can impair the immune response to HCC therapies, potentially impacting overall survival [47]. Additional factors include the HIV *Tat* gene, which directly influences the biology of uncontrollably occurring tumors [70]. The *Tat* gene prompts the expression of numerous transcription factors, growth factors, and multiple cytokines while encouraging cell growth and preventing apoptosis [70].

Torgersen et al. indicated that the increased HIV RNA and prolonged HIV viremia persistence also contribute to HCC development [40]. HIV viremia accelerates the progression of liver fibrosis to cirrhosis or may directly promote the development of hepatocarcinogenesis through oxidative stress, immune dysregulation, and a reduction in CD4+ cells, which leads to microbial translocation [40,71,72]. Given the impact of HIV-related chronic inflammation and immune dysregulation in hepatocarcinogenesis, Kim et al. analysed inflammation-associated DNA methylation patterns and demonstrated their potential as biomarkers for early HCC detection in HIV-positive individuals [48].

Several studies indicated that HIV can also result in cancer when it co-exists with viral hepatitis [43,69,73]. This was confirmed in a study by Maponga et al., which showed that among the 64% HBV-positive HCC patients that were enrolled in their study, about 27% were previously found to be HIV co-infected [49]. This means that the co-infection might have led to a weaker immune system, resulting in the spread of cancerous cells in the liver. Studies also suggest that in most cases of HIV and viral hepatitis co-infection, the leading cause of HCC is the development of cirrhosis [40,49,74]. These pathogenic agents induce acute hepatic inflammation, which may indicate an increased possibility of hepatocyte neoplastic transformation, which could potentially progress to cirrhosis and HCC [5]. Co-infection of HIV and HCV is linked to an impaired sustained virologic response [75]. This effect is caused by a reduced ability of the immune system to respond to HCV infection and an increased stimulation of hepatic stellate cells as a result of liver damage, thereby raising the levels of pro-inflammatory cytokines [76]. Giannini et al. demonstrated that persistent HCV infection increases mortality risk, whereas achieving sustained virologic response improves survival [58]. According to a study by Lee et al. [60], sustained virological response can be achieved through direct-acting antiviral therapy, which significantly improves the overall survival of HCV-related HCC patients, regardless of their disease stage [64]. A study by Surguladze et al. reported that older individuals and women with HCV infection exhibit a higher risk of developing liver cancer [57].

Infections with HBV and HCV can result in inflammatory processes, destruction of cells, and hepatic exudation. In a study by Yan et al., it was shown that these conditions are strongly linked to Th17 cell stimulation, which is also linked to a high viral burden and the proliferation of hepatic macrophages in the body [55]. Th17 cells secrete IL-17, IL-21, and IL-22, which are cytokines that can worsen the state of inflammation by stimulating additional inflammatory mediators and leukocytes that accumulate at inflammation sites. Combined, these conditions can lead to the development of cancer [55]. Choi et al. demonstrated that a higher baseline HBV viral load is significantly associated with an increased risk of HCC, suggesting that early viral suppression might be crucial in reducing long-term liver cancer risk [56].

Yang et al. reported that HBV infection can induce genetic mutations as a result of its ability to integrate its DNA into the host genome. In their study, they discovered that the HBV DNA integration breakpoints can be dispersed randomly throughout the human chromosomes and that roughly 61.86% of these integration occurrences can be found in gene-coding regions, which then proves that HBV has a direct hepatocarcinogenic impact [41]. A study by Dai et al. also suggests that a high HBV DNA level independently increases the risk of tumor recurrence in HBV-related HCC patients [59].

Other studies indicate that another carcinogenic factor of HBV is its encoded proteins, with the HBx protein being especially significant [23,26]. This protein may regulate apoptosis, block intracellular signalling pathways, and promote the development of HCC through epigenetic modifications [77]. This was also elucidated in a study by Shi et al., where they found the HBx protein to notably downregulate the long non-coding RNA (lncRNA) OIP5-AS1, which serves as a tumor suppressor in HBV-positive HCC patients. The HBx protein repressed the expression of OIP5-AS1 through the inhibition of a transcriptional factor, peroxisome proliferator-activated receptor α (PPARα) [63]. A study by Zhang et al. indicated that the HBx gene also exhibits several single-nucleotide polymorphisms (SNPs) in the HBV genome that are associated with the progression of HCC over time [64].

A study by Deng et al. reported that other risk factors of HCC in hepatitis patients include the elevated levels of Pentraxin 3 (PTX3), an inflammatory protein, which they found to be significantly higher in the serum of HBV patients than healthy individuals [52]. Clinical research has shown that serum PTX3 is a reliable diagnostic indicator of fibrosis in persistent HCV infection, which may be linked to an increased risk of HCC development in chronic HCV infection [52,78]. One study by Wang et al. also showed another risk factor of HCC in HBV-infected individuals to be the hypomethylated MDM2 promoter, where it is stated that its gene products contribute significantly to HCC and encourage tumor growth. They revealed that individuals with HBV-related HCC exhibit MDM2 promoter hypomethylation [53]. The combination of MDM2 promoter hypomethylation and alpha-fetoprotein (AFP) enhances the diagnosis of HBV-associated HCC [53]. When McMahon and colleagues investigated variables connected to the progression of HCV infection leading to the development of HCC, they found HCV genotype 3 to be the most significant variable. In their findings, they discovered that people with HCV genotype 3 infection had a higher chance of dying from HCC, end-stage liver disease, and liver-related causes [54]. A study by Wu et al. also revealed that HBV-positive HCCs exhibit greater genomic instability, with higher scores for copy number amplifications, deletions, homologous recombination deficiency, and intratumor heterogeneity [62].

Studies reveal that viral hepatitis can also be associated with the development of HCC when it co-exists with schistosomiasis [79,80,81]. There are still no clear biomolecular explanations for the connection between schistosoma infection and HCC [81]. However, studies indicate that schistosoma impacts viral persistence via T-helper-2 polarization, which may lead to increased viral load, leading to the elevated risk of HCC development [32,80]. A study by AlGabbani indicates that people with chronic schistosomiasis are susceptible to PIK3CA (phosphoinositide-3-kinase-catalytic-alpha) gene mutations, which can eventually cause liver cancer and hepatocyte fibrosis [38]. When genetic damage increases, the progression of the malignant features of hepatocytes also increases, leading to the evolution of phenotypic and genetic variations of HCC [30,82]. This can mean that the PIK3CA gene mutations in chronic schistosomiasis patients may be predictive of HCC [38].

Roderfeld and colleagues demonstrated that the cytotoxic elements secreted from parasite eggs in organs and tissues cause the proto-oncogenes c-Jun and STAT3 to become permanently activated, meaning they will promote uncontrolled cell division and prevent the natural process of apoptosis, allowing abnormal cells to survive and multiply [32]. STAT3 and c-Jun are transcription factors or proto-oncogenes that, when they are stimulated, facilitate the onset and advancement of dysplasia during the tumorigenesis of HCC [32,83]. A study by von Bulow et al. illustrated that *S. mansoni* eggs utilize the host environment for nutrient acquisition via metabolic rearrangement of enterocytes and hepatocytes [84]. The metabolic rearrangement in hepatocytes might include alterations in lipid metabolism or inflammation-related pathways, contributing to the formation of immune cell clusters around the eggs [37,84]. This leads to liver damage, including cirrhosis, and eventually liver cancer. When eggs land in the liver, they activate oxidative stress, which then damages DNA, possibly leading to hepatocellular malignancy [84]. Therefore, oxidative stress is a constant threat to hepatocytes throughout infection, and this stress can cause DNA damage that could lead to cancer [65,84].

## 5. Conclusions

This review collected literature that focuses on the association of three infections, HIV, viral hepatitis, and schistosomiasis, with the development of HCC. Literature has revealed that HIV impairs immunity, increasing a person’s vulnerability to other infections and complications. Although HIV does not directly cause HCC, it can accelerate the development of liver disease when it co-exists with HBV or HCV, the two main known risk factors for HCC. Chronic HBV and HCV may result in liver inflammation and damage, which increases the chances of developing malignant tumors over time. Long-term schistosomiasis infection increases the risk of HCC by causing liver fibrosis, portal hypertension, and cirrhosis. The interactions between schistosomiasis and viral hepatitis can exacerbate liver impairment and accelerate the progression to HCC. However, there is limited research on the correlation between schistosomiasis and HCC, and there are no studies that have examined the effect of the triple infection with HIV, viral hepatitis, and schistosomiasis on the progression of liver cancer. Further research on the impact of triple infection on the development of liver cancer is needed to emphasize the need for coordinated efforts, particularly where schistosomiasis, as a disregarded infection, is prevalent and more likely to overlap with other infectious diseases such as hepatitis and HIV.

## 6. Study Limitations

In this systematic review, several limitations were identified. One major challenge was the scarcity of research specifically addressing the association between schistosomiasis and liver malignancy, limiting the ability to draw firm conclusions in this area. Additionally, although numerous studies explored the effect of HIV, viral hepatitis, and schistosomiasis, they were frequently conducted in relation to co-infections with other diseases rather than specifically addressing liver cancer. Importantly, no studies were identified that evaluated the combined impact of these three infections (HIV, viral hepatitis, and schistosomiasis) on liver cancer, creating a significant gap in understanding their potential synergistic effects. These limitations highlight the need for more targeted prospective research on both individual and combined impacts of these infections on liver cancer.

## Figures and Tables

**Table 1:** Summary of the most relevant articles included in this study based on HIV association with liver cancer.

Research Title	Aim	Methodology	Study Region and Model (In Vivo/In Vitro/Human Model)	Clinical Findings	Ref.
Risk factors for liver cancer in HIV endemic areas of Western Kenya	To identify possible risk factors for HCC and to explore the association between HIV infection and HCC.	- CD4+ T lymphocytes were measured by flow cytometry. - Serum Alpha-fetoprotein levels were quantified for the detection of HCC. - Aflatoxins in urine were detected by ELISA techniques.	- Africa, Kenya - Human and in vitro study - A case-control study; (*n* = 257 HCC patients)	- HIV-positive patients had a higher risk of HCC (33.5%) than those who were HIV-negative (10.9%). - The combination of HIV with either HBV or HCV further increased the risk of developing liver cancer.	[5]
The impact of HIV infection on clinical presentation and mortality among persons with hepatocellular carcinoma in Kampala, Uganda	To evaluate how HIV infection influences the outcomes and presentation of HCC.	- A complete blood cell count, HIV, and HBV antibody and antigens detection were performed using laboratory testing. - HCC survival rate was assessed using the Kaplan–Meier curves.	- Africa, Uganda - Human and in vitro study - A case-control study; (*n* = 441 HCC patients)	- 87% died within 12 months of HCC diagnosis. - 18% cases were HIV-positive. - A CD4 count of less than 200 cells per cubic milliliter was associated with a higher mortality rate.	[2]
HIV RNA, CD4+ percentage, and risk of hepatocellular carcinoma by cirrhosis status.	To determine whether reduced CD4+ cell percentage and increased or long-term HIV viremia were linked to HCC, with a focus on whether this risk is modified by the presence of cirrhosis.	- Participants were categorized based on their cirrhosis status, and the risk of developing HCC was evaluated across these groups. - Cox regression was employed to identify the hazard ratios of HCC.	- North America, United States - Human study - A retrospective cohort study (*n* = 35,695 HIV-positive).	- A lower CD4+ percentage was also linked to a higher risk of HCC, with the association being more pronounced in those with cirrhosis.	[40]
Influence of HIV-related immunodeficiency on the risk of hepatocellular carcinoma	To investigate HIV-associated immunosuppression as a potential risk factor for HCC in HIV-positive individuals.	- Lymphocyte count and CD4+ cell count were conducted using flow cytometry techniques. - Odds ratios were calculated by conditional logistic regression.	- Europe, Switzerland - Human and in vitro study - A case-control study (*n* = 26 HCC patients).	- The risk of HCC was significantly increased by 70% in individuals with CD4+ counts below 200 cells/µL compared to those with higher counts.	[42]
The risk of hepatocellular carcinoma among individuals with acquired immunodeficiency syndrome in the United States	To investigate the incidence of HCC and additional hepatobiliary malignancies in AIDS-affected individuals.	- Incidence Rate Ratios (RR) among AIDS patients were computed by Poisson’s regression. - The risk of HCC in relation to the overall population was assessed through standardized incidence ratios (SIRs).	- North America, United States - Human study - A retrospective cohort study (*n* = 615,150 AIDS patients).	- 366 cases of HCC among individuals with AIDS were identified. - The risk of HCC in individuals with AIDS was nearly four times higher than in the general population.	[43]
Serum protein biomarkers for HCC risk prediction in HIV/HBV co-infected people: a clinical proteomic study using mass spectrometry	To identify the expression of DEPs (differentially expressed proteins) associated with HCC in order to filter out potential biomarkers in the evaluation of HCC in HIV/HBV co-infected individuals.	- Serum protein changes in HCC and non-HCC patients co-infected with HIV and HBV were investigated through liquid chromatography-tandem mass spectrometry. - Gene Ontology and Disease Ontology enrichment analysis were performed on the DEPs.	- Asia, China - Human and in vitro study - An observational study (*n* = 13 HCC patients).	- 124 DEPs were identified, 25 were upregulated, and 29 were downregulated. - A total of 11 proteins were selected as the major DEPs associated with HCC.	[44]
Incidence of hepatocellular carcinoma in hepatitis C cirrhotic patients with and without HIV infection: a cohort study	To measure the prevalence of HCC in cirrhotic HCV patients regardless of their HIV status during the HAART era.	- HCV was confirmed by the detection of HCV RNA as well as plasma anti-HCV antibody. - HIV was tested by Western Blot. - HCC was detected through ultrasound screening.	- South America, Argentina - Human and in vitro study - A prospective cohort study (*n* = 148).	- HCC developed in 12 participants (5 co-infected as well as 7 single-infected). - HIV co-infection does not increase the incidence of HCC in patients with HCV-related cirrhosis during the HAART era.	[45]
Integrated phenotyping of the anti-cancer immune response in HIV-associated hepatocellular carcinoma.	To illustrate the functional features of the T cell infiltrate of HIV-linked HCC	- Multiplex immunostaining for PD-1 and CD4+ was performed. - PD-L1 expression was achieved in tumor-infiltrating T lymphocytes. - Sequencing for T-cell receptors was conducted through the immunoSEQ assay. - Targeted transcriptomic profiling was conducted using the NanoString PanCancer Immune panel.	- Europe, United Kingdom - Human and in vitro study - A retrospective cohort study (*n* = 63 HIV associated HCC patients).	- In HIV-associated HCC, cytotoxic T cells were found to be more dysfunctional. - An increased expression of PD-1 in HIV-associated HCC patients was observed. - A gene subset evaluation showed dysregulated innate and adaptive immunity in HIV-positive cases.	[46]
Retrospective analysis of patients with hepatocellular carcinoma complicated with human immunodeficiency virus infection after hepatectomy.	To evaluate the survival outcomes and clinical effectiveness of hepatectomy in patients with HCC co-existing with HIV infection.	- 27 patients went for a liver resection, and 29 were on non-surgical therapy. - General outcome data were analysed, and risk factors were determined.	- Asia, China - Human study - A retrospective cohort study (*n* = 56 HCC/HIV patients).	- The surgical group had a significantly longer overall survival compared to the non-surgical group. - The risk factors in the surgical group included the lower CD4+ count and postoperative microvascular invasion.	[47]
Cell-free DNA methylation-based inflammation score as a marker for hepatocellular carcinoma among people living with HIV	To evaluate the potential of a cell-free DNA methylation-based inflammation score as a biomarker for detecting HCC in people living with HIV.	- Inflammation-DNAm score and HCC association were evaluated using multivariable logistic regression. - ROC analysis was performed to compare the ability of the DNAm score against alpha-fetoprotein (AFP).	- North America, United States - Human and in vitro study - (*n* = 249 HIV patients).	- An elevated inflammation-DNAm score was linked to a 29% higher likelihood of developing HCC. - The DNAm score showed enhanced effectiveness in differentiating HCC cases from controls.	[48]

**Table 2:** Summary of the most relevant articles included in this study based on viral hepatitis association with liver cancer.

Research Title	Aim	Methodology	Study Region and Model (In Vivo/In Vitro/Human model)	Clinical Findings	Ref.
Hepatitis B virus-associated hepatocellular carcinoma in South Africa in the era of HIV.	To characterize the natural progression of HCC in individuals infected with HBV alone, as well as those co-infected with HBV and HIV.	- Hepatitis and HIV were tested using immunoassay kits. - DNA quantification and genotyping were performed on HBV-positive samples. - HCC survival rate after diagnosis was assessed using the Kaplan–Meier method.	- Africa, South Africa - Human and in vitro study - A prospective cohort study (*n* = 107 HCC patients).	- 64% were HBV-positive, and 22% were HIV-positive. - Among the HBV-positive cases, 27% were HIV co-infected. - HIV-infected individuals, especially women, developed HCC at a younger age than those who were HIV-negative.	[49]
Hepatitis C virus genotype affects survival in patients with hepatocellular carcinoma.	To determine whether a person’s survival rate from HCV-associated HCC was influenced by their HCV genotype.	- Survival analyses were conducted using the Kaplan–Meier method. - A Cox proportional hazards model was employed for univariate and multivariate analyses.	- Asia, South Korea - Human study - A retrospective cohort study (*n* = 180 HCV-related HCC patients).	- Patients with HCV genotype 2 had better survival outcomes, with a significantly lower mortality rate (37.2%) compared to those with genotype 1 (57.0%) and genotype 3 (68.8%).	[50]
Differential serum cytokine profiles in patients with chronic hepatitis B, C, and hepatocellular carcinoma.	To investigate and compare serum cytokine profiles among patients with chronic HBV, chronic HCV, and HCC to identify cytokine patterns associated with disease progression and potential biomarkers for HCC.	- Multiplex analysis (Luminex 200 IS) was utilized to determine the serum concentrations of 51 cytokines. - Univariate and multivariate analyses were performed to assess the relationships between cytokine profiles and disease status.	- North America, United States - Human and in vitro study - A prospective cohort study (*n* = 411 chronic liver disease patients).	- Specific cytokines, including IL-6 and IL-8, might serve as potential biomarkers for distinguishing HCC from chronic hepatitis conditions. - Alterations in cytokine profiles could be linked to disease progression from chronic hepatitis to HCC.	[51]
Serum pentraxin 3 as a biomarker of hepatocellular carcinoma in chronic hepatitis B virus infection.	To assess whether serum pentraxin 3 (PTX3) quantification could enhance HCC diagnosis in cases of persistent HBV infection.	- Serum HBV DNA was assessed through HBV qPCR. - HBV antibodies were identified by ELISA. - Serum AFP was determined by Automated Eleceyes. - PTX3 concentration in serum was determined using an immunoassay.	- Asia, China - Human and in vitro study - A case-control study (*n* = 516 HBV patients).	- Significantly higher levels of PTX3 were found in HBV patients compared to healthy controls (*p* < 0.001), and in HCC patients compared to those with chronic hepatitis (*p* < 0.001). - PTX3 was a distinct risk variable for HCC.	[52]
Combined use of murine double minute-2 promoter methylation and serum AFP improves diagnostic efficiency in hepatitis B virus-related hepatocellular carcinoma.	To investigate the diagnostic significance of methylation of the murine double minute-2 (MDM2) promoter in patients with HBV-correlated HCC	- MDM2 promoter’s methylation level was determined through Methylation-specific PCR. - Quantitative real-time PCR was used to verify the MDM2 expression levels. - Tumor necrosis factor-α (TNF-α) as well as interleukin-6 (IL-6) concentrations in plasma were measured using ELISA.	- Asia, China - In vitro and human study - A cross-sectional study (*n* = 168 patients: 100 HCC, 31 liver cirrhosis, 37 HBV).	- Individuals with HBV-related HCC exhibit MDM2 promoter hypomethylation. - The combination of MDM2 promoter methylation and alpha-fetoprotein (AFP) enhanced the diagnosis of HBV-associated HCC.	[53]
Infection with hepatitis C virus genotype 3 is an independent risk factor for end-stage liver disease, hepatocellular carcinoma, and liver-related death.	To investigate variables connected to the progression of HCV infection.	- Real-time polymerase chain reaction was used to identify the HCV virus genotype. - Hazard ratios were calculated using the Cox proportional hazards model.	- North America, United States - Human and in vitro study - A retrospective cohort study (*n* = 1080 chronic HCV patients).	- People with HCV genotype 3 infection had a higher chance of dying from HCC, end-stage liver disease, and liver-related causes.	[54]
Prevalence and clinical relevance of T-helper cells, Th17 and Th1, in hepatitis B virus-related hepatocellular carcinoma.	Assessing the immune status of Th1 and Th17 cells among individuals having HBV-associated and non-HBV-associated HCC.	- Samples of tissues and blood were collected. - Th17 and Th1 cell distributions as well as phenotypic characteristics were identified using immunohistochemistry and flow cytometry.	- Asia, China - In vitro and human study - A retrospective cohort study (*n* = 150 HCC patients).	- Th17 and Th1 cell counts were noticeably higher in the tumors of individuals with HCC.	[55]
Chronic hepatitis B baseline viral load and on-treatment liver cancer risk: A multinational cohort study of HBeAg-positive patients	To confirm the relationship between baseline HBV viral load and the risk of HCC during treatment in a larger cohort	- All patients had a baseline HBV viral load of ≥5.00 log10 IU/mL, and the risk of HCC was assessed based on baseline viral load.	- Asia and Oceania - Human study - A multinational cohort study (*n* = 7545 HBeAg-positive patients)	- Baseline HBV viral load was significantly linked to HCC risk, even with antiviral treatment. - Participants with the highest baseline viral loads who subsequently initiated treatment exhibited the lowest long-term risk of HCC development.	[56]
Hepatitis C virus attributable liver cancer in the country of Georgia, 2015–2019: a case–control study.	To evaluate the contribution of HCV infection to the development of HCC in the Georgian population.	- Age- and sex-stratified and bivariate logistic regression analyses were used to evaluate the association between HCV and liver cancer.	- Europe, Georgia - Human study - A case-control study (*n* = 3874)	- older individuals and women infected with HCV had a higher likelihood of developing both liver cancer and HCC. - HCV infection was strongly associated with an increased risk of developing liver cancer and HCC	[57]
Absence of Viral Replication Is Associated With Improved Outcome in Anti-HCV-Positive Patients With Hepatocellular Carcinoma	To assess the impact of active HCV infection on the survival of patients undergoing treatment for HCC.	- HCV-positive HCC patients were categorized based on an active HCV infection or a sustained virological response (SVR). - Differentiations were conducted using propensity score matching (PSM) to account for clinical, demographic, and oncological characteristics.	- Europe, Italy - Human study - (*n* = 3123 HCV-positive HCC patients)	- Patients who achieved sustained virologic response (SVR) had a longer median overall survival after HCC treatment compared to those with an active HCV infection. - Active HCV infection was linked to a higher risk of mortality.	[58]
Preoperative Antiviral Therapy and Long-Term Outcomes for Hepatitis B Virus-Related Hepatocellular Carcinoma After Curative Liver Resection: A Multicenter Analysis	To evaluate the impact of preoperative antiviral therapy (AVT) on tumor recurrence and overall survival in HBV-related HCC patients undergoing curative hepatectomy.	- Short-term outcomes, clinical features, overall survival, and time-to-recurrence were compared. - Multivariate Cox regression analysis was used to evaluate the data.	- Asia, China - Human study - A retrospective cohort study (*n* = 565 patients)	- A high preoperative HBV DNA level independently increased the risk of tumor recurrence. - Preoperative AVT for over a year was associated with improved overall survival and a lower incidence of tumor recurrence by reducing the preoperative HBV DNA levels.	[59]
Direct-Acting Antiviral Therapy for Patients with HCV-Related Hepatocellular Carcinoma: A Nationwide Cohort Study.	To investigate the effect of Direct-Acting Antiviral (DAA) therapy on the overall survival of patients with HCC through a nationwide cohort study.	- A database of adults undergoing DAA therapy for HCV, excluding individuals with other viral infections or end-stage HCC, was analysed. - The adjusted odds ratio for sustained virological response and the adjusted hazard ratio for overall survival were then calculated.	- Asia, Taiwan - Human study - A nationwide cohort study (*n* = 2205 HCC patients)	- For the HCC group, the SVR rates were 96.6% and 98.8% for the control group (*p* < 0.001). - The presence of HCC is an independent risk factor influencing SVR outcomes.	[60]
Contribution of hepatitis B virus and hepatitis C virus to liver cancer in China’s northern areas: experience of the Chinese National Cancer Center.	To ascertain how HBV and HCV affect the development of primary liver cancer (PLC) in the northern regions of China	- Serum HBsAg, anti-HBc, and anti-HCV were identified using microparticle immunoassay and electrochemiluminescence immunoassay. - Quantification was performed using qRT-PCR. - Gene sequencing and genotype analysis were conducted on a subset of patients diagnosed with HCC.	- Asia, China - In vitro and human study - A retrospective cohort study (*n* = 2172 primary liver cancer patients).	- From the cohort, 83.9% had HCC. Among the HCC cases, HBV was found in 86.0% cases, HCV was found in 2.5%, and HBV + HCV co-infection was found in 6.7% of cases. - This showed evidence that individuals with HCC have an active replication of HBV and HCV.	[61]
A comprehensive comparison of molecular and phenotypic profiles between hepatitis B virus (HBV)-infected and non-HBV-infected hepatocellular carcinoma by multi-omics analysis	To perform an extensive comparison between HCC patients with and without HBV infection using multi-omics analyses to identify molecular and phenotypic differences that could potentially explain the distinct pathogenesis and clinical outcomes between these two groups.	- A multi-omics analysis, which included genomics, transcriptomics, proteomics, and metabolomics data, was conducted. The data was analysed through advanced bioinformatics tools.	- Asia, China - Human study - A retrospective study (*n* = 10 HCC patients: 5 HBV positive HCC, 5 HBV negative HCC)	- HBV-infected HCC patients had poorer overall survival rates compared to their non-HBV-infected counterparts. - HBV-infected HCC patients exhibited greater genomic instability, increased activation of DNA repair and immune-related pathways, decreased activation of stromal and oncogenic signalling pathways, and demonstrated improved outcomes with immunotherapy.	[62]
Hepatitis B virus X protein promotes tumor glycolysis by downregulating lncRNA OIP5-AS1/HKDC1 in HCC	To explore the role of the HBV X protein (HBx) in promoting glycolysis in HCC and to investigate how HBx affects the expression of the long non-coding RNA OIP5-AS1 and its downstream target, HKDC1, a key glycolytic enzyme.	- HCC cell lines were cultured and transfected with HBx-expressing plasmids. - qRT-PCR and western blotting techniques were used to measure the expression levels of lncRNA OIP5-AS1 and HKDC1. - Glycolytic function in the HCC cells was evaluated by measuring glucose uptake, lactate production, and ATP levels.	- Asia, China - In vitro and human study - A cross-sectional study (*n* = 40 HCC patients: 20 HBV negative and 20 HBV positive)	- HBx was shown to reduce the expression of lncRNA OIP5-AS1 in HCC cells. - Following OIP5-AS1 knockdown, glucose consumption and lactate production were both significantly reduced in SNU-449 and Huh7 cells, suggesting suppressed glycolysis in these cells.	[63]
Detection of HBV DNA integration in plasma cell-free DNA of different HBV diseases utilizing a DNA capture strategy.	To detect the integration of HBV DNA into the host genome by analysing plasma cell-free DNA (cfDNA) in patients with different HBV-related diseases.	- cfDNA was extracted from the plasma samples. - A DNA capture strategy was employed to enrich HBV DNA fragments from cfDNA. - Next-generation sequencing (NGS) was used to analyze the enriched HBV DNA and identify integration sites in the host genome. - The frequency, distribution, and characteristics of HBV DNA integration sites were analyzed and compared across different disease groups.	- Asia, China - In vitro and human - A cross-sectional study (*n* = 25: 9 HBV, 10 cirrhosis, and 6 HCC).	- Patients with HCC exhibited the highest frequency of HBV DNA integration. - The number of unique HBV integration sequences was significantly higher in HCC patients compared to HBV and liver cirrhosis patients, suggesting a greater integration rate in the advanced stages of liver disease.	[41]
The dynamic variation position and predominant quasispecies of hepatitis B virus: Novel predictors of early hepatocarcinoma.	To discover new predictors for early HCC by examining the dynamic variation positions and dominant quasispecies of the HBV through the use of advanced sequencing techniques.	- Second-generation sequencing (NGS) and high-order multiplex droplet digital PCR (ddPCR) were used to examine HBV quasispecies in serum samples. - Non-synonymous single-nucleotide polymorphisms (SNPs) and their variant proportions were detected before HCC onset.	- Asia, China - In vitro and human study - A retrospective cohort study (*n* = 247 HBV carriers).	- 15 non-synonymous SNPs with higher variant proportions were identified in HCC cases, with key variations found predominantly in the HBX gene (46.7%) compared to other genes (HBS 12.7%, and HBC 13.8%). Specific mutations and their combinations were more prevalent in early HCC cases, suggesting their potential as predictive markers for early HCC.	[64]

**Table 3:** Summary of the most relevant articles included in this study based on schistosomiasis association with liver cancer.

Research Title	Aim	Methodology	Study Region and Model (In Vivo/In Vitro/Human Model)	Clinical Findings	Ref.
Mutations in the TP53 and PIK3CA genes in hepatocellular carcinoma patients are associated with chronic Schistosomiasis.	To assess the PIK3CA gene’s genetic variation as well as the histopathological alterations in the liver tissue of individuals suffering from long-term schistosomiasis in order to forecast HCC.	- PCR and sequencing were used to analyze mutations in the TP53 and PIK3CA genes	- Asia, Saudi Arabia - Human and in vitro study - A retrospective study (*n* = 20: 9 schistosomiasis + ve, 11 schistosomiasis -ve).	- People who have chronic schistosomiasis are susceptible to PIK3CA gene mutations, which can eventually cause liver cancer and hepatocyte fibrosis.	[38]
*Schistosoma mansoni* Egg–Secreted Antigens Activate Hepatocellular Carcinoma–Associated Transcription Factors c-Jun and STAT3 in Hamster and Human Hepatocytes.	To look at the hepatocellular stimulation of STAT3 and c-Jun caused by an infection with *Schistosoma mansoni*.	- In the liver of hamsters infected with *S. mansoni*, immunohistochemistry, western blotting, and the electrophoretic mobility-shift assay were used to analyze the function and expression of c-Jun and STAT3. - Hepatocellular stimulation of c-Jun was indicated by increased phosphorylation (Ser73) and translocation.	- Europe, Germany - In vivo and in vitro study - A retrospective experimental study.	- The study found that the stimulation of HCC proto-oncogenes c-Jun and STAT3 by compounds secreted by tissue-trapped schistosome eggs is a significant factor in the development of liver cancer in individuals infected with *S. mansoni*.	[32]
*Schistosoma mansoni*–Induced Oxidative Stress Triggers Hepatocellular Proliferation.	To examine whether the effects of oxidative stress cause hepatocellular proliferation following infection with *S. mansoni*.	- Western blotting, immunohistochemistry, and qRT-PCR were used to examine the replication stress response, proliferation, and the cell cycle.	- Europe, Germany - In vivo and in vitro study - A retrospective experimental study.	- The study found that the oxidative stress brought on by *S. mansoni* eggs triggers hepatocellular proliferation.	[65]
Hepatosplenic schistosomiasis-associated chronic portal vein thrombosis: risk factor for hepatocellular carcinoma?	To determine the prevalence of HCC as well as portal vein thrombosis (PVT) in schistosomiasis patients	- PVT was identified through the Doppler ultrasonography on the portal system. - HCC was initially identified by ultrasonography and subsequently verified by axial imaging, abdominal CT, or MRI.	- South America, Brazil - Human study - A retrospective cohort study (*n* = 126).	- PVT was seen in 73 (57.9%) of the 126 schistosoma cases. - HCC was detected in six (8.1%) of the 73 PVT cases.	[30]

## Data Availability

No new data were created or analyzed in this study. Data sharing is not applicable to this article.

## Supplementary Materials

The following supporting information can be downloaded at: https://www.mdpi.com/article/10.3390/microorganisms13122753/s1, Table S1: PRISMA 2020 checklist, Table S2: The Effective Public Health Practice Project (EPHPP) Quality Assessment Tool. Ref. [85] is included in Supplementary Materials.
