# Supplementary material for: HIV, Viral Hepatitis, and Schistosomiasis Association with Liver Cancer: A Systematic Review
Source: Microorganisms. 2025 Dec 4;13(12):2753. doi: 10.3390/microorganisms13122753 (PMC12735182; doi:10.3390/microorganisms13122753)
Supplement: Supplementary file 1 [file microorganisms-13-02753-s001.zip › Table S2. Quality assessment- K Canham.pdf]

The Effective Public Health Practice Project (EPHPP) Quality Assessment Tool.

Rating key:

	**STRONG**	**MODERATE**	**WEAK**	**NOT APPLICABLE**
**RATING**	**1**	**2**	**3**	**-**

Global rating for the acceptance of studies:

Strong: At least four components rated strong - Accepted

Moderate: Fewer than four strong ratings, and only one weak rating- Accepted

Weak: Two or more components rated weak – Not Accepted

Quality assessment for studies summarized in Table 1:

Study ID	Selection Bias	Study Design	Confounders	Blinding	Data Collection Methods	Withdrawals & Dropouts	Analysis	Global Rating
Otedo et al., 2018 (5)	2	2	1	3	1	2	1	2
Nsibirwa et al., 2023 (2)	2	2	1	3	1	1	1	1
Togersen et al., 2020 (40)	2	1	1	3	1	2	1	1
Clifford GM et al., 2008 (42)	2	2	1	3	1	2	1	2
Sahasrabuddhe VV et al., 2012 (43)	2	1	2	2	1	2	2	2
Ke H et al., 2023 (44)	2	2	2	3	1	-	2	2

Di Benedetto N et al., 2014 (45)	2	1	2	3	1	2	2	2
Pinato DJ et al., 2023 (46)	2	1	2	3	1	-	1	2
Lu J-J et al., 2024 (47)	2	2	2	3	2	-	1	2
Kim K et al., 2024 (48)	2	2	1	3	1	-	1	2

Quality assessment for studies summarized in Table 2:

**Study ID**	**Selection Bias**	**Study Design**	**Confounders**	**Blinding**	**Data Collection Methods**	**Withdrawals & Dropouts**	**Analysis**	**Global Rating**
Maponga TG et al., 2020 (49)	2	2	2	3	2	2	2	2

Park HK et al., 2019 (50)	2	1	1	3	2	2	1	2
Estevez J et al., 2017 (51)	1	2	2	3	1	-	2	2
Deng H et al., 2020 (52)	1	1	2	3	1	-	1	1
Wang J-W et al., 2020 (53)	2	2	2	3	1	-	2	2
McMahon BJ et al., 2017 (54)	1	1	1	3	1	1	1	1
Yan J et al., 2014 (55)	2	2	2	3	1	2	1	2
Choi W-M et al., 2024 (56)	1	1	1	3	1	2	1	1
Surguladze S et al., 2024 (57)	2	2	2	3	1	2	1	2
Giannini EG et al., 2025 (58)	1	1	1	3	1	2	1	1

Dai M-G et al., 2024 (59)	2	2	2	3	1	2	1	2
Lee S-W et al., (60)	1	1	1	3	1	1	1	1
Wang MinJie WM et al., 2017 (61)	2	2	-	3	1	-	2	2
Wu Z et al., 2024 (62)	2	2	2	3	1	-	1	2
Shi F et al., 2024 (63)	2	2	2	3	1	-	1	2
Yang Z et al., 2024 (41)	2	2	2	3	1	-	1	2
Zhang C et al., 2024 (64)	2	2	2	3	1	-	1	2

Quality assessment for studies summarized in Table 3:

Study ID	Selection Bias	Study Design	Confounders	Blinding	Data Collection Methods	Withdrawals & Dropouts	Analysis	Global Rating
AlGabbani Q, 2022 (38)	2	2	2	3	2	-	2	2
Roderfeld M et al., 2020 (32)	2	1	2	3	1	-	1	2
Von Buelow V et al., 2024 (65)	2	2	2	3	1	-	1	2
Darce GFB et al., 2023 (30)	2	2	2	3	2	2	2	2
